# Characterization of methicillin resistant *Staphylococcus Aureus* in municipal wastewater in Finland

**DOI:** 10.1016/j.onehlt.2024.100881

**Published:** 2024-08-21

**Authors:** Ahmad Ibrahim Al-Mustapha, Ananda Tiwari, Venla Johansson, Viivi Heljanko, Lehto Kirsi-Maarit, Anssi Lipponen, Sami Oikarinen, Tarja Pitkänen, Annamari Heikinheimo, WastPan Study Group

**Affiliations:** a Department of Food Hygiene and Environmental Health, Faculty of Veterinary Medicine, University of Helsinki, Finland; b Department of Veterinary Services, Kwara State Ministry of Agriculture and Rural Development, Ilorin, Kwara State, Nigeria; c Department of Veterinary Public Health and Preventive Medicine, Faculty of Veterinary Medicine, University of Ibadan, Oyo State, Nigeria; d Finnish Institute for Health and Welfare, THL, Department of Health Security, Kuopio, Finland; e Tampere University, Faculty of Medicine and Health Technology, Tampere, Finland; f Finnish Food Authority, Ruokavirasto, Seinäjoki, Finland

**Keywords:** Antimicrobial resistance, *Staphylococcus aureus*, Antibiotic resistance gene, *LukS/F-PV*, Wastewater based surveillance

## Abstract

Wastewater-based surveillance (WBS) of multidrug-resistant bacteria could complement clinical data, serving as a population-level early warning tool. This study evaluated WBS as a pandemic preparedness tool, by selectively isolating and culturing methicillin-resistant *Staphylococcus aureus* (MRSA) with CHROMagar MRSA. Some 24-h composite wastewater samples (*n* = 80) were collected from ten treatment plants across Finland between February 2021 and January 2022. MRSA prevalence in wastewater samples was 27.5% (*n* = 22/80), showing seasonal and temporal variations. Phenotypic antimicrobial susceptibility testing (AST) with microdilution showed that over 80% of isolates were drug-resistant to clindamycin, sulfamethoxazole/trimethoprim, tetracycline, fusidic acid, and erythromycin. Four isolates (18.2%) were vancomycin-resistant. WGS revealed that 31.8% (*n* = 7) of the isolates belonged to the ST8-*t*008 and ST6-*t*304 *spa* types, respectively. In addition, two *spa* types (*t*011 and *t*034) belong to the CC398 complex. The *mec*A gene was found in all isolates (*n* = 22) and three tetracycline resistance determinants (*tet*38, *tet*K, and *tet*M) were detected with *tet*38 being the most abundant (81.8%, *n* = 18/22). Three isolates harboured the plasmid-mediated *sat*4 gene that confers resistance to Streptothricin. In addition, resistance determinants to macrolide antibiotics (*mph* (C)/*msr* (A) and fosfomycin (*fos*B) were detected in the seven isolates that belonged to *spa* type *t*008. All isolates except one harboured the SCC*mec_*type_IVa(2B). Six ST8 isolates harboured the LukS/F-PV genes encoding the Panton–Valentine leukocidin (PVL) and were also positive for the Arginine Catabolic Mobile Element (ACME), suggesting they belong to the USA300 clone. The Inc18 plasmid was the most abundant as it was detected in 72.7% (*n* = 16/22) of the isolates. Other plasmid replicons detected were the rep_trans and repA_N which were detected in 45.4% (*n* = 10/22) and 40.9% (*n* = 9/22) of the isolates respectively. Ten isolates harboured at least three plasmid replicons and no plasmid replicons were detected in four isolates (ST6/*t*304). The cgMLST revealed that some isolates aggregated into two genomically indistinguishable clusters: ST6/*t*304 belonging to cluster type CT12405 (≤20 allelic differences) and ST8/*t*008 belonging to cluster type CT1925 (<8 allelic differences). Overall, we found a high genotypic concordance with the national clinical bacterial resistance data. Our study demonstrates the sensitivity of culture-based wastewater surveillance for MRSA using clinical media following pre-enrichment, reliably predicting pathogen occurrence at the population level.

## Graphical abstract

Culture-based approach to the detection of methicillin-resistant *staphylococcus aureus* in wastewater samples in Finland.

**Figure:**
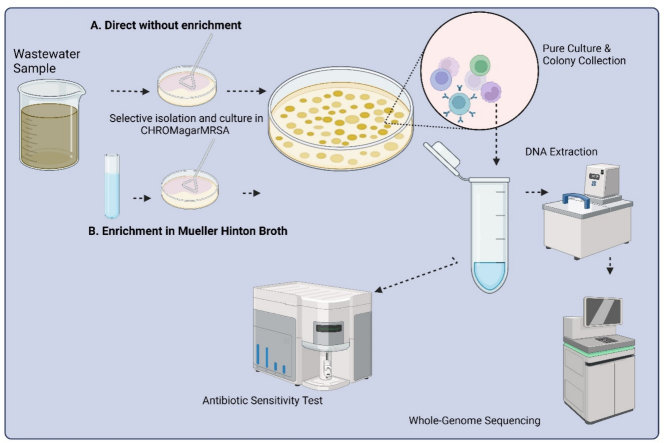

## 1 Introduction

Antimicrobial resistance (AMR) is a global public health threat and infection due to antibiotic-resistant bacteria (ARB) alone caused ∼1.27 million (95% UI 0.911–1.71) deaths in 2019 [1]. Of these, Methicillin Resistant *S. aureus* (MRSA) accounted for over 100,000 deaths globally [1], posing a major therapeutic hurdle among Gram-positive bacterial pathogens [2,3]. MRSA is listed as a notifiable disease in Finland and other Nordic countries [4,5]. There is increasing concern about the growing resistance of pathogenic bacteria in the environment, particularly evidenced by the uptick in MRSA cases, especially in the Nordic countries [[6], [7], [8], [9]].

To tackle the AMR challenge, a Global Action Plan (GAP) was instituted to limit the emergence and spread of these ARBs, improve their detection, and protect antibiotics and the environment through prudent antimicrobial usage and the development of antimicrobial stewardship programs [10]. One of the key components of the GAP on AMR was the need to improve the evidence-based data on AMR. To achieve this, it is essential to have relatively cheap and sensitive data at the population level.

Wastewater-based surveillance (WBS) has emerged as a promising strategy for population-level epidemiological surveillance, providing objective and comprehensive analysis, as well as nearly real-time monitoring of the circulation of targeted pathogens in a given population [11]. This is because wastewater contains a wide range of biological materials from human excretion spanning faeces, urine, mucus, sputum, and skin lesions from early stages of colonization to different further stages of infections by personal care, irrespective of the development of clinical symptoms activity [12]. However, WBS cannot provide evidence at an individual level, and it mostly complements existing clinical surveillance systems by providing a representative community sample, data for communities where clinical data is scanty or unavailable, and acts as an early warning system to provide population-level data on disease trends [10]. Generally, MRSA received more attention in clinical epidemiology than in wastewater studies, probably due to their lower detection rates in wastewater [3,5]. For instance, Meir-Gruber et al., [3] reported the low occurrence of MRSA in municipal sewage in Israel and a study from Australia reported that MRSA isolates were occasionally detected in 24% of the WWTPs and 5.33% of the samples during 2017–2019 [13].

While MRSA prevalence in Finland has generally remained stable, its incidence in the hospital districts of southwest Finland increased from 24.1 to 31.2 cases/100000 persons/year between 2007 and 2016 [9]. The increasing threat of ARB mandated the inclusion of important pathogenic bacteria including MRSA in the WASTPAN [14]. The project explored the potential of wastewater surveillance to serve as a tool for monitoring spatial and temporal trends and changes in these pathogens in Finland. This will enable the monitoring of ethically less-binding samples from humans, their companion animals, and their immediate environments and it offers a good cost-effective matrix to survey entire cities for fluctuations in antibiotic-resistant bacteria (ARBs) and antibiotic resistance genes (ARGs) [[14], [15], [16], [17]]. This paper used a culture-based approach to explore the viability of wastewater samples as a reliable source of epidemiological information at a population level. Furthermore, we provided epidemiological insights by comparing isolates with annual clinical MRSA reports and finally explored the genomic diversity of these isolates. We anticipate this study to offer insights into the spatiotemporal distribution of MRSA in Finland.

## 2 Materials and methods

### 2.1 WWTP data, study settings, and sampling

A total of 80 composite wastewater samples (1 l each) were collected from influents in ten wastewater treatment plants (WWTPs) across Finland. Eight monthly samples were collected from each of the selected WWTPs between February 2021 and January 2022, as a part of the WASTPAN project [14]. The samples were aseptically collected and immediately transported by ice packs to the bacteriology Laboratory of the Department of Food Hygiene and Environmental Health at the University of Helsinki for further analysis.

### 2.2 Isolation of MRSA isolates

We compared two isolation techniques for the cultural isolation of MRSA from composite wastewater samples: Direct plating and pre-enrichment. The former was done by directly pouring 1 ml of wastewater on CHROMagar MRSA agar plates (Paris, France), a clinical chromogenic medium, whereas the latter included a pre-enrichment step. This step involved the selective enrichment of MRSA in the samples by incubating five millilitres of wastewater sample in 45 ml of Mueller Hinton Broth supplemented with 6.5% NaCl and incubated for 18–24 h at 37 °C. Then, a loopful (10 μl) of the overnight medium was streaked onto a CHROMagar MRSA (Paris, France), for the selection and differentiation of MRSA. Throughout this paper, we refer to pre-enriched samples as those enriched for MRSA in Mueller Hinton Broth before culturing on CHROMagar MRSA agar plates. Presumptive MRSA isolates were sub-cultured on fresh CHROMagar plates and finally purified on nutrient agar (Oxoid, Basingstoke, UK). One to three colonies were picked from each plate and were further reliably identified (best match score value of >2.300) using the Matrix-Assisted Laser Desorption Ionization Time of Flight - Mass Spectrometry (MALDI-ToF) (Bruker, Bremen, Germany) following the Biotyper protocol.

### 2.3 Antibiotic susceptibility testing

The broth microdilution assay was used to test the antibiotic susceptibility profiles of the pre-enriched isolates against ten classes of antibiotics contained in the EUSTAPF Sensititre plates (Thermo Scientific, Vantaa, Finland). The EUSTAPF Sensititre plates contained the following antibiotics: Cefoxitin; Ceftarolin; Clindamycin; Daptomycin, Erythromycin, Fusidic acid; Gentamicin, Levofloxacin, Linezolid, Moxifloxacin, Mupirocin, Norfloxacin, Rifampin; Teicoplanin, Telavancin, Tetracycline, Tobramycin; Trimethoprim/Sulfamethoxazole, and Vancomycin. Based on the epidemiological cut-off values (ECOFF), isolates were classified as resistant or susceptible [18]. Phenotypically, an isolate was designated as multidrug-resistant (MDR) if it confers resistance to at least three classes of antibiotics, and extensively drug resistance (XDR) if it confers resistance to at least five different classes of antibiotics [19]. The MRSA strain AF1214–14 was used as the positive control whereas *S. aureus* strain ATCC 12600 was used as a negative control.

### 2.4 Whole genome sequencing of MRSA

The total DNA of the isolates was extracted using the commercially available DNeasy blood and tissue Kit (Qiagen, Hilden, Germany) in a QIACUBE Connect (Qiagen, Hilden, Germany) following the manufacturer's instructions. The output purified DNA was quantified with a Qubit Fluorometer 4.0 (Invitrogen, Singapore). DNA samples were purified using a Qiagen Purification kit and the sequencing libraries were prepared using the Nextera Flex Kit according to the manufacturer's instructions. Then, we conducted whole genome sequencing (WGS) of all MRSA isolates using the Illumina HiSeq platform using the 2 × 200 paired-end read approach (Illumina Inc., San Diego, CA, USA). The raw sequencing read output had its adaptor trimmed using Trimmomatic v. 0.36 [20]. and the quality of the reads was assessed using the FastQC tool v.0.12.0. The raw reads were assembled into contigs using SKESA v.2.4.0 [21]. The sequence raw reads are openly available at the ENA under the project ID: PRJEB73878 and individual accession numbers are available in supplementary Table S1.

### 2.5 Analysis of whole genome sequence data

Analysis of the assembled contigs was done after parsing them into the Ridom SEQSPHERE+ *v. 7.7.5* bacterial WGS analytical pipeline (Ridom GmbH, Münster, Germany). The NCBI AMR Finder Plus tool was used to identify ARGs in the isolates [22]. Three tools from the CGE analytical pipeline were used for the identification of the plasmid replicons [23], virulence-associated genes [24], and the SCC mec elements respectively [25] using a 90% identification threshold and 60% minimum length. The RIDOM Seqsphere+ pipeline was also used to perform the core genome Multi Locus Sequence Typing (cgMLST) against 1861 targets for gene-by-gene allele calling to investigate the genetic relatedness of the assembled genomes. The pipeline generates a cgMLST complex type (CT) which was designed to identify isolates with very similar profiles. A cluster alert distance of 20 allele differences and a cluster alert quality threshold of at least 90% good cgMLST targets were used to detect closely related isolates [26].

## 3 Results

**Fig. 1:**
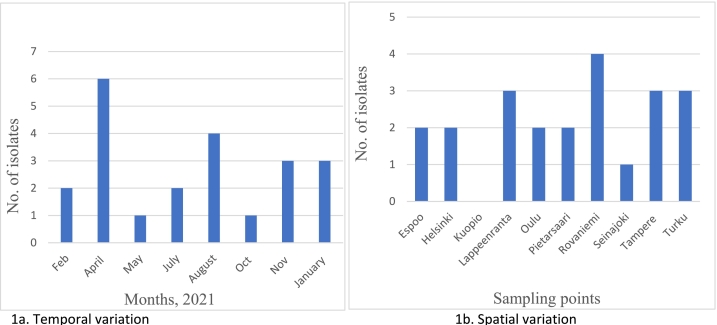
Variations in occurrence of MRSA in wastewater across Finland.

Among the two culture methods (direct culture and pre-enriched), the pre-enriched method yields significantly more MRSA isolates. The direct plating yielded only nine MRSA isolates (11.3%) whereas 22 of the 80 (27.5%) were obtained from the pre-enriched wastewater samples. All further analyses and interpretations were based on the isolates obtained from the pre-enriched samples (*n* = 22). There were seasonal variations in the occurrence of MRSA in wastewater as the highest isolation rate (75%, *n* = 6/8) was in early spring (April) (Fig. 1a). Isolation of MRSA was highest in wastewater from Rovaniemi (50%, *n* = 4/8) which was followed by the detection of three MRSA isolates (37.5%, *n* = 3/8) each from Lappeenranta, Tampere, and Turku respectively. The isolation rate from other cities is shown in Fig. 1b.

### 3.1 Phenotypic antibiotic susceptibility testing

Phenotypic AST showed that some MRSA isolates were extensively drug-resistant (resistant to at least 5 classes of antibiotics). Altogether, 20 isolates (91%) were resistant to clindamycin, tetracycline, and daptomycin (Table 1). Similarly, there was high resistance to fusidic acid (86.2%, *n* = 19), and erythromycin (82%, *n* = 18). Four isolates (18.2%) expressed vancomycin resistance whereas two isolates (9.1%) expressed phenotypic resistance to telavancin (a glycopeptide antibiotic) and ceftaroline (a fifth-generation cephalosporin) respectively.

**Table 1:**
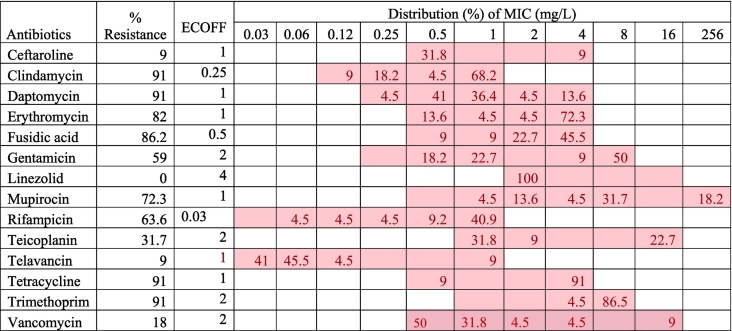
Phenotypic antimicrobial susceptibility testing of methicillin-resistant *Staphylococcus aureus* isolated from wastewater (*n* = 22).

The highlighted field showed the range of antibiotic dilutions available in the EUSTAPF sensititre plates. MIC = Minimum inhibitory concentration, ECOFF = epidemiological cut-off value.

### 3.2 Molecular typing of MRSA isolates

Molecular typing of the MRSA isolates revealed that the 22 isolates belonged to eight different spa types (Table 2, Supplementary Table S2). Eight of the isolates (36.4%) belonged to the spa type *t*304 and were the most abundant whereas seven isolates (31.8%) belonged to spa type *t*008. In addition, our results showed that two *spa* types (*t*011 and *t*034) belonging to the CC398 complex were detected in wastewater samples. No spa type was predominant in any of the cities as the four isolates from Rovaniemi belonged to three spa types: *t*008 (*n* = 2), *t*127 (*n* = 1), and *t*026 (*n* = 1). All isolates harboured the staphylococcal cassette chromosome *mec* (SCC*mec*) type IVa(2B).

**Table 2:** Comparison of *spa* typing of Methicillin-Resistant *S. aureus* isolated from wastewater in Finland against the 2021 Infectious Diseases Report (FINRES).

Spa type	CC	Number of isolates (%)	2021 Infectious Diseases Report [27]
*t*008	CC8	31.8% (*n* = 7)	11%
*t*304	CC5	31.8% (*n* = 7)	10%
*t*127	CC1	9.1% (*n* = 2)	5%
*t*026	CC45	9.1% (*n* = 2)	<1%
*t*172	–	4.6% (*n* = 1)	5%
*t*011	CC398	4.6% (*n* = 1)	<1%
*t*034	CC398	4.6% (*n* = 1)	<1%
*t*5526	CC5	4.6% (*n* = 1)	<1%

CC-Clonal complex.

Six of the MRSA isolates (AA31, AA47, AA49, AA57, AA64, and AA72) harboured the *LukF-PV* and *lukS-PV* genes that code the virulence factor-Panton–Valentine leukocidin (PVL), a two-component toxin that lyses phagocytic leucocytes. Other leukocidins (coded by the *lukD* and *lukE* genes) were detected in 50% of the isolates (*n* = 11). These six isolates also harboured the Arginine Catabolic Mobile Element (ACME) which plays a role in bacterial virulence and transmission and contains an arginine deiminase (arc) pathway and an oligopeptide permease (opp-3) system. This suggests that the isolates belonged to the USA300 clone (PVL and ACME positive CA-MRSA). Other virulence-associated genes detected included several other toxin genes (*hla, hlgA*, *hlgB*, *hlgC, sea*), host immune genes (*sak* and *scn),* and exoenzyme genes (*aur, spl*A, *spl*B, *spl*E).

### 3.3 Antibiotic resistance genes

**Fig. 2:**
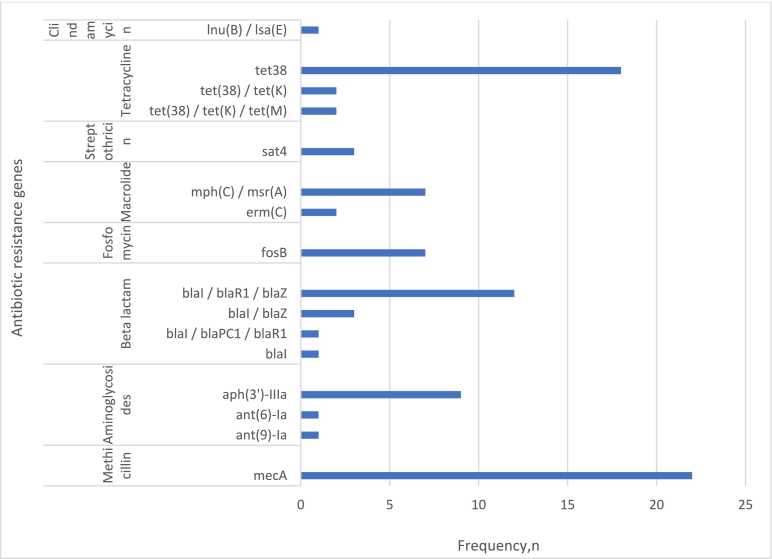
Diversity of antibiotic resistance genes in MRSA isolates (*n* = 22) from wastewater in Finland.

There was a high concordance between the phenotypic and genotypic antibiotic resistance patterns of the isolates. The WGS analysis revealed that the MRSA isolates harboured several antibiotic-resistance genes. The *mec*A gene was found in all isolates (*n* = 22). Resistance to tetracycline was conferred by three genes (*tet*38, *tet*K, and *tet*M) with tet38 being the most abundant (81.8%, *n* = 18/22). Resistance to Beta-lactam antibiotics was conferred by *bla*I (4.5%, *n* = 1), a combination of *bla*I, *bla*PC1, and *bla*R1 (4.5%, *n* = 1), as well as by a combination of *bla*I, *bla*R1, and *bla*Z in 54.5% (*n* = 12) of the isolates. Three resistance genes confer resistance to aminoglycosides (*ant*(9)-Ia, *ant*(6)-Ia, and *aph*(3′)-IIIa (Fig. 2). The *sat*4 gene, a plasmid-mediated streptothricin acetyltransferase that confers resistance to Streptothricin (a nucleoside antibiotic) was detected in 13.6% (*n* = 3/22) of the isolates. In addition, resistance determinants to macrolide antibiotics (*mph*(C) / *msr*(A - erythromycin) and phosphonic antibiotics (*fos*B -Fosfomycin) were detected in the seven isolates that belonged to spa type *t*008. Another macrolide resistance gene (*erm*(C) was detected in two isolates that belonged to spa type *t*127. A single isolate (spa type *t*034) harboured the *Inu*(B) / *Isa*(E) that confers resistance to lincosamide antibiotics (clindamycin). None of the plasmid-mediated or previously reported point mutations in the ribosomal proteins (L3, L4, and L22) or 23S rRNA operon associated with linezolid resistance were detected in the sequences.

### 3.4 Plasmids

A total of five plasmid replicons were detected in the MRSA isolates. The Inc18 plasmid was the most abundant as it was detected in 72.7% (*n* = 16/22) of the isolates. Other plasmid replicons detected were the rep_trans and repA_N which were detected in 45.4% (*n* = 10/22) and 40.9% (*n* = 9/22) of the isolates respectively. The rep3 and repL plasmids were detected in 31.8% (*n* = 7) and 4.5% (*n* = 1) respectively. Ten isolates harboured at least three plasmid replicons and no plasmid replicons were detected in four isolates (ST6/*t*304) (Supplementary Table S2).

### 3.5 Core genome MLST

**Fig. 3:**
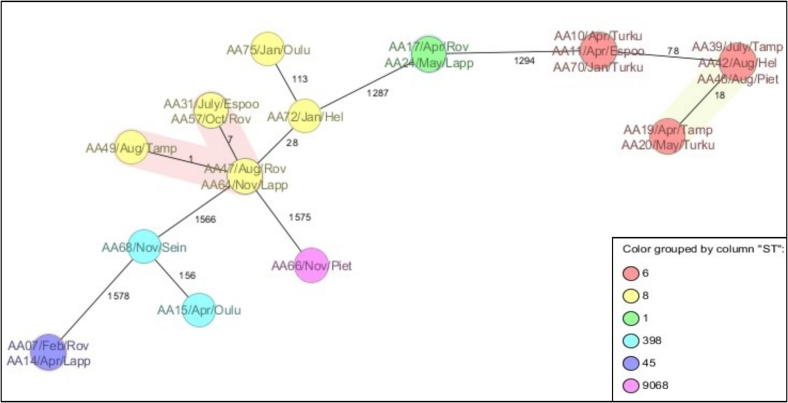
Core genome MLST of the isolates showing clustering of MRSA isolates (*n* = 22). Hel-Helsinki, Lapp – Lappeenranta, Rov – Rovaniemi, Sein – Seinajoki, Tamp – Tampere. All samples were collected between February 2021 to Jan 2022. The numbers represent the allelic differences between isolates. The isolates were denoted with isolate ID, month, and city of origin. Each colour represented a sequence type (ST).

The MLST of the isolates revealed that the 22 MRSA isolates belonged to five known STs with 36.4% (*n* = 8/22) and 31.8% (*n* = 7/22) of the isolates belonging to ST6 and ST8 respectively. Two isolates each belonged to ST1, ST45, and ST398 respectively whereas an isolate (AA66) was assigned to ST9068 by PubMLST. The core genome MLST (based on 1604 of the 1861 genes) of the isolates revealed that isolates aggregated into five clusters with some isolates having no allelic differences (Fig. 3). The minimum spanning tree showed that five isolates from four different cities (Helsinki, Pietarsaari, Tampere, and Turku) belonging to the same ST6/*t*304 had <20 allelic differences and belonged to CT 12405. With <8 allelic differences, isolates from Espoo, Rovaniemi, Lappeenranta, and Tampere clustered together (ST8/*t*008/CT 1925) and were therefore genomically indistinguishable. Two closely related and indistinguishable isolates clustered in Rovaniemi and Lappeenranta (ST1 and ST45).

## 4 Discussion

Our findings revealed that wastewater surveillance can be used as a predictive tool to obtain population-level data on MRSA. This is evidenced by the high concordance between the spa type obtained from WW surveillance and the Infectious Diseases Register in Finland. We believe that culture-based approaches are more amenable and more compatible with traditional clinical human health risk assessments than other approaches such as metagenomic deep sequencing which requires more computational power, analytical algorithms, extensive training, and is more expensive [28,29]. Detection of *t*008/ST8 and *t*304/ST6, the two more prevalent spa types in clinical samples (blood, pus, etc.) across Finland further buttresses the sensitivity of the culture-based approach to WW surveillance. Our findings were similar to the Finnish Infectious Disease report which showed that over the last decade, there has been an increase in t304 and a decrease in the *t*172 spa types across Finland [27]. In addition, the detection of t011 and t034 belonging to the CC398 complex in wastewater could be attributed to animal sources, contact with animal reservoirs, and possible zoonotic transmission to humans [30]. The spa types identified in this study were also the most reported in clinical samples (blood, pus, etc.) in Finland. The identification of the same MRSA clone in different cities indicated the clonal dissemination of MRSA in Finland (Fig. 3).

Our findings revealed that MRSA was detected in 27.5% of the wastewater samples. Despite the different sample types (wastewater vs clinical samples), our finding can be compared to the findings of a 10-year review of total MRSA cases in hospital districts of southern Finland in which the authors reported that 32% of all cases were community-associated [9]. The study further reported a 7.5% increase per year in the total number of cases between 2007 and 2016. Based on the data, one-third of the CA-MRSA cases were traced to immigrants, refugees, and residents of foreign countries [9].

The AST profile of the MRSA isolates revealed that all the isolates were MDR. There was high phenotypic resistance to clindamycin, sulfamethoxazole/trimethoprim, tetracycline, fusidic acid, and erythromycin. These findings are contrary to the findings of the 2020 FINRES report which reported that only 2.4% of the 2176 MRSA isolated from clinical cases (blood and pus specimens) were MDR [27]. These differences could be attributed to the different sampling types (wastewater vs clinical), sampling size, and the differences between population and hospital-based samples. Also, antibiotics and other co-selection factors in sewage form a persistent selection pressure for the emergence and dissemination of ARGs and ARBs in wastewater, especially by horizontal gene transfer (HGT) [[31], [32], [33], [34]]. In-vitro resistance of MRSA isolates to telavancin, a reserve antibiotic against complicated skin and skin structure infections (CSSSIs), and other antibiotics in the WHO reserve list such as ceftaroline and daptomycin further emphasizes the need to preserve antibiotics and explore the genomic epidemiology of these pathogens [35].

The diverse resistance phenotypes observed in the MRSA isolates were attributed to the diverse ARGs that were detected during WGS. All of the isolates harboured the *mec*A gene which conferred resistance to methicillin, penicillin, and other beta-lactam antibiotics [36]. The *mecA* gene encodes an alternative penicillin-binding protein, *PBP2a*. It is part of a 21- to 60-kb staphylococcal chromosome cassette *mec* (SCC*mec*), a mobile genetic element that may also contain genetic structures such as Tn*554*, pUB110, and pT181 which encode resistance to non-β-lactam antibiotics [3,36]. In addition to the *mec*A gene, resistance determinants to beta-lactam antibiotics were mostly conferred by *bla*Z (with only a few isolates harbouring the *bla*I, blaPC1, and blaR1 genes). For some antibiotics, the phenotypic resistance could not be linked with a resistance gene. More studies are needed to ascertain whether MRSA strains in wastewater acquire more durable cell wall structures, that could mechanically lower the susceptibility against tested antimicrobials. Also, such phenotypic resistance to antibiotics could be attributed to a multiplicity of other resistance mechanisms including target modification, enzymatic drug inactivation, and decreased antibiotic uptake or efflux [[37], [38]]. For instance, macrolide resistance determinants in MRSA have been attributed to the modification of the bacterial ribosome by erm-gene-encoded 23S rRNA methyltransferase [38]. The MRSA isolates did not contain the MLS_B_ gene which confers resistance to macrolides, lincosamides, and Streptogramins. Nine isolates however carried the macrolide resistance determinants [*mph*(C), *msr*(A), and *erm*(C)] which could confer cross-resistance to lincosamides.

Although seven MRSA isolates (spa type *t*008 only) harboured the *fos*B gene which is a resistance determinant for Fosfomycin, the *tet*38 gene, a chromosomally encoded efflux transporter of *S. aureus* also acts as an efflux transporter of fosfomycin [39]. The high occurrence of the aminoglycoside resistance determinant, *aph*(3′)-IIIa was similar to other studies [40,41]. The *sat*4 gene, a plasmid-mediated resistance determinant for Streptothricin has also been reported in clinical MRSA isolates from Germany [42].

Although wastewater treatment plants significantly reduce the microbial diversity of ARBs and ARGs, they do not completely remove all ARBs and ARGs posing potential risks to the downstream receiving environment [[43], [44], [45], [46], [47]], or even amplify some genes via HGT [48]. Hence, the occurrence of ARGs in wastewater poses the risk of environmental contamination. Hence, wastewater monitoring presents an opportunity for environmental surveillance and would help inform quantitative and qualitative microbial risk assessments, which is critical to guiding regulatory limits for their discharge, and for public safety [49].

Transfer of conjugative plasmids is one of the major mechanisms of transmission of ARGs between bacteria [28,47]. The Inc18 broad-host conjugative plasmid has been shown to encode a variety of antibiotic-resistant determinants, including those that confer resistance to vancomycin, chloramphenicol, and the macrolide-lincosamide-streptogramin (MLS) group of antibiotics [50]. The four isolates which harboured no plasmid replicon had a smaller number of ARGs compared to those that had any of the plasmid replicons. Hence, we could suggest that some of the ARGs were carried on these putative plasmid replicons. This is similar to the findings of Mores et al., which detected several classes of plasmid replicon proteins (rep3, repA_N, and rep_trans) in clinical MRSA isolates in Egypt [51].

The MRSA isolates belonged to diverse STs and spa types, but all belonged to the mec-IVa(2B) element. The SCC_mec_Iva(2B) has been globally associated with community-acquired MRSA (CA-MRSA). In addition, the positivity for the LukF/S-PV gene is commonly considered a CA-MRSA marker [52]. PVL-positive strains have been reported to be responsible for abscess formation, tissue necrosis, and increased inflammatory responses [53]. As all of the ST8 PVL-positive isolates also harboured the ACME locus which was previously reported to increase bacterial virulence, fitness, and transmission, they could be assigned to the USA300 clone; a predominant community-associated strain of MRSA in North America [54]. In addition, PVL/ACME-positive ST8 strains of MRSA were previously reported in Japan [55], Belgium [56], England [57], and Slovakia [58] outside of its prevalent region of North America. Several of the virulence-associated genes that were detected in the isolates are involved in staphylococcal food poisoning, play a significant role in the host-pathogen interaction, help the pathogen to evade the host's immune response, and in biofilm formation [[59], [60], [61]].

Globally, the emergence and spread of ARB have raised serious public health concerns. Many studies have highlighted the role of wastewater as a significant environmental reservoir of AMR as it represents an ideal environment for pathogens and antimicrobial-resistant genes to persist and proliferate [31,32,[45], [46], [47], [48], [49]]. Across Europe, WBS has been used to track infectious diseases in the population including the polio virus, SARS-CoV-2 or chemical agents such as illicit drug use [33]. In Finland, WBS have been used in monitoring *Citrobacter freundii*, and carbapenemase-producing Enterobacteriaceae [62,63]. Currently, there is no consensus on the ideal detection protocols to identify, quantify, and determine the source of the pathogens for WBS. Of the several approaches, the culture-based approach is relatively cheaper, sensitive, and can be feasible even in resource-limited settings. This method is the gold standard for AST and for assessing the relationship between genotypic and phenotypic characteristics. In addition, our findings were in agreement with previous studies that reported the seasonality of MRSA infections with a higher burden in warm seasons [64,65].

Despite the strong correlation between the culture-based WBS of MRSA and the clinical bacterial resistance report in Finland, there are some limitations to its utility as an early warning tool. Firstly, a low level of infection in a community might affect the bacterial concentration and make it very difficult to isolate, but we found pre-enrichment step significantly increases the detection sensitivity. Secondly, the interpretation of wastewater surveillance data obtained in communities that are not served by municipal sewer systems might be inconclusive and difficult. Thirdly, it is difficult to determine the source of some of the molecular features (ARGs, plasmids, bacteriophages) of MRSA isolates as these features could have been acquired anywhere along the wastewater pipeline (environment) and not from the population of a community. Finally, wastewater system design and operations, such as pretreatment of incoming wastewater, can affect test results. Despite these limitations, wastewater surveillance could help overcome known limitations of clinical surveillance such as low population coverage, high cost, as well as testing and reporting delays. The methodology could be further improved by studying larger sample sizes, a larger volume of samples, or the filtration of samples.

## 5 Conclusion

This study reports the WBS for MRSA at a population level and our findings were concordant with the Finnish infectious diseases report. The culture-based WBS approach is still the gold standard method for ARB in clinical settings. The information obtained from culture-based methods with clinical media has proved to be a useful complementary tool to reliably predict the occurrence of MRSA in Finland. This would provide evidence-based information needed for public health decision-making and preparedness plans. Our findings revealed that pre-enrichment increased the diagnostic sensitivity and detection of MRSA isolates in wastewater. The isolates harboured several ARGs, virulence-associated genes, and plasmids that could pose a risk of environmental contamination. Further research in areas of comparative whole genome-based genomic studies of human and wastewater MRSA isolates could provide more detailed information on these pathogens.

## Funding

This study formed a part of the WastPan consortium project, funded by the Research Council of Finland (formerly the Academy of Finland) (grant numbers: 1339415, 1339416, 1339417), the Finnish Government, the Ministry of Agriculture and Forestry Finland, the Ministry of the Environment Finland, the Finnish Water Utilities Development Fund and the National Emergency Supply Agency Finland.

## Ethics approval

Not required.

## CRediT authorship contribution statement

**Ahmad Ibrahim Al-Mustapha:** Writing – review & editing, Writing – original draft, Validation, Project administration, Formal analysis, Data curation, Conceptualization. **Ananda Tiwari:** Writing – review & editing, Writing – original draft, Validation, Project administration, Formal analysis, Data curation. **Venla Johansson:** Writing – review & editing, Validation, Methodology, Formal analysis. **Viivi Heljanko:** Writing – review & editing, Visualization, Methodology, Formal analysis. **Lehto Kirsi-Maarit:** Writing – review & editing, Supervision, Resources, Project administration, Methodology. **Anssi Lipponen:** Writing – review & editing, Validation, Resources, Project administration. **Sami Oikarinen:** Writing – review & editing, Validation, Resources, Project administration. **Tarja Pitkänen:** Writing – review & editing, Validation, Supervision, Resources, Project administration, Investigation. **Annamari Heikinheimo:** Writing – review & editing, Writing – original draft, Supervision, Resources, Methodology, Formal analysis, Conceptualization.

## Declaration of competing interest

The authors declare that the research was conducted in the absence of any commercial or financial relationships that could be construed as a potential conflict of interest. Kirsi-Maarit Lehto and Sami Oikarinen are the stakeholders of GreenSeq Ltd. Finland. All claims expressed in this article are solely those of the authors and do not necessarily represent those of their affiliated organizations or those of the publisher, the editors, or the reviewers.

## Data Availability

The sequence raw reads are openly available at the ENA under the project ID: PRJEB73878. Individual accession numbers are available in supplementary Table S1.

## Appendix A Supplementary data

Supplementary material. Image 1
