# Supplementary material for: Characterization of methicillin resistant Staphylococcus Aureus in municipal wastewater in Finland
Source: One Health. 2024 Aug 21;19:100881. doi: 10.1016/j.onehlt.2024.100881 (PMC11388770; doi:10.1016/j.onehlt.2024.100881)
Supplement: Supplementary file 1 — Supplementary material. [file mmc1.docx]

**Characterization of Methicillin Resistant *Staphylococcus Aureus* in Municipal Wastewater in Finland**

Ahmad Ibrahim Al-Mustapha, ^1,2,3 *^ Ananda Tiwari,^1,4^ Venla Johansson,^1^ Viivi Heljanko, ^1^ Lehto Kirsi-Maarit, ^1,5^ Anssi Lipponen,^4^ Sami Oikarinen, ^5^ Tarja Pitkänen, ^1,4^ Annamari Heikinheimo,^1,6^ and WastPan Study Group

**Supplementary files**

**Table S1. Sequencing parameters of wastewater MRSA isolates (n=22).**

| Sample ID | Time of collection | City of collection | Avg. Coverage | Approximated Genome Size (Mbases) | N50 | Contig Count | Accession Number |
| --- | --- | --- | --- | --- | --- | --- | --- |
| AA07 | Feb-21 | Rovaniemi | 110 | 2.8 | 459141 | 66 | ERR13511555 |
| AA10 | Apr-21 | Turku | 116 | 2.8 | 426186 | 53 | ERR13511556 |
| AA11 | Apr-21 | Espoo | 117 | 2.8 | 426230 | 52 | ERR13511557 |
| AA14 | Apr-21 | Lappeenranta | 111 | 2.8 | 359204 | 61 | ERR13511558 |
| AA15 | Apr-21 | Oulu | 123 | 2.7 | 13656 | 459 | ERR13511559 |
| AA17 | Apr-21 | Rovaniemi | 111 | 2.8 | 326437 | 30 | ERR13511560 |
| AA19 | Apr-21 | Tampere | 112 | 2.8 | 446268 | 28 | ERR13511561 |
| AA20 | May-21 | Turku | 112 | 2.8 | 446209 | 26 | ERR13511562 |
| AA24 | May-21 | Lappeenranta | 110 | 2.8 | 326202 | 35 | ERR13511563 |
| AA31 | Jul-21 | Espoo | 117 | 2.9 | 66181 | 184 | ERR13511564 |
| AA39 | Jul-21 | Tampere | 99 | 2.8 | 426257 | 37 | ERR13511565 |
| AA42 | Aug-21 | Helsinki | 114 | 2.8 | 496437 | 36 | ERR13511566 |
| AA46 | Aug-21 | Pietarsari | 113 | 2.8 | 496480 | 34 | ERR13511567 |
| AA47 | Aug-21 | Rovaniemi | 108 | 2.9 | 345783 | 46 | ERR13511568 |
| AA49 | Aug-21 | Tampere | 109 | 2.9 | 346346 | 49 | ERR13511569 |
| AA57 | Oct-21 | Rovaniemi | 108 | 2.9 | 345764 | 47 | ERR13511570 |
| AA64 | Nov-21 | Lappeenranta | 109 | 2.9 | 346341 | 46 | ERR13511571 |
| AA66 | Nov-21 | Pietarsari | 113 | 2.8 | 169893 | 51 | ERR13511572 |
| AA68 | Nov-21 | Sienajoki | 111 | 2.8 | 424006 | 47 | ERR13511573 |
| AA70 | Jan-22 | Turku | 115 | 2.8 | 426185 | 52 | ERR13511574 |
| AA72 | Jan-22 | Helsinki | 112 | 2.9 | 345764 | 50 | ERR13511575 |
| AA75 | Jan-22 | Oulu | 117 | 2.8 | 295437 | 64 | ERR13511576 |

**Table S2. Molecular characteristics of MRSA isolated from wastewater in Finland.**

| Isolate ID (City of isolation) | Direct plating | Pre-enrichment | Antimicrobial resistance pattern | PVL | Spa type | CC | ST | SCC-mec element | ARGs | Point mutations  Associated with AMR | Plasmids |
| --- | --- | --- | --- | --- | --- | --- | --- | --- | --- | --- | --- |
| AA07 (Rovaniemi) | - | + | CLI, TRM, TET, DAP, FUS, GEN, MUP, RIF, TEI, | Nil | *t*026 | CC45 | 45 | SCCmec_type_IVa(2B) | tet(38), blaI / blaZ, mecA | dfrB, fusA, gyrA, grlB, ileS, 23S | Inc18 (rep6 & repUS5) |
| AA10 (Turku) | - | + | CLI, FUS, | lukD, lukE | *t*304 | CC5 | 6 | SCCmec_type_IVa(2B) | tet(38), mecA | fusA, gyrA, 23S | Nil |
| AA11 (Espoo) | - | + | CLI, TRM, TET, CPT, DAP, ERY, FUS, GEN, MUP, RIF, TEI, VAN | lukD, lukE | *t*5526 | CC5 | 6 | SCCmec_type_IVa(2B) | tet(38), blaI, blaZ, mecA | gyrA, grlB, ileS, dfrB, pbp2, 23S, | Inc18, repA_N, rep3 (rep5a) |
| AA14 (Lappeenranta) | - | + | TRM, | Nil | *t*026 | CC45 | 45 | SCCmec_type_IVa(2B) | tet(38), blaI / blaZ, mecA | fusA, gyrA, 23S | Inc18 |
| AA15 (Oulu) | + | + | CLI, TRM, TET, FUS, MUP, RIF | Nil | *t*011 | CC398 | 398 | SCCmec_type_IVa(2B) | tet(38), tet(K), tet(M), blaI, mecA | dfrB, fusA, gyrA, grlB, 23S | rep_trans (rep7a, repUS43) |
| AA17 (Rovaniemi) | - | + | CLI, TRM, TET, DAP, ERY, FUS, GEN, RIF | LukD, lukE | *t*127 | CC1 | 1 | SCCmec_type_IVa(2B) | tet(38), tet(K), blaI/blaR1, blaZ, mecA, sat4, ant(6)-Ia, aph(3')-IIIa, erm(C) | dfrB, fusA, gyrA, ileS, pbp2, pbp4 | Inc18, rep3, rep_trans (rep7a, repUS7c), repL |
| AA19 (Tampere) | + | + | CLI, TRM, TET, DAP, ERY, FUS, GEN, TEI, | LukD,lukE | *t*304 | CC5 | 6 | SCCmec_type_IVa(2B) | tet(38), mecA | dfrB, fusA, gyrA, ileS, pbp2, pbp4 | Nil |
| AA20 (Turku) | - | + | CLI, TRM, TET, ERY, FUS, | lukD, lukE | *t*304 | CC5 | 6 | SCCmec_type_IVa(2B) | tet(38), mecA | dfrB, fusA, gyrA, ileS, pbp2, pbp4, 23S | Nil |
| AA24 (Lappeenranta) | - | + | CLI, TET, DAP, ERY, FUS, | lukD, lukE | *t*127 | CC1 | 1 | SCCmec_type_IVa(2B) | tet(38), tet(K), blaI/blaR1, blaZ, mecA, aph(3')-IIIa, erm(C) | dfrB, fusA, gyrA, pbp2 | Inc18, rep3, repL, rep_trans |
| AA31 (Espoo) | - | + | CLI, TRM, TET, ERY, FUS, GEN, MUP, RIF, VAN | lukD, lukE,lukF, lukS-PV | *t*008 | CC8 | 8 | SCCmec_type_IVa(2B) | tet(38)blaI / blaR1 / blaZ, mecA, aph(3')-IIIa, msr(A), mph(C) | dfrB, fusA, grlA, gyrA, ileS, pbp2, pbp4, | Inc18, repA_N, rep_trans(repUS7c) |
| AA39 (Tampere) | + | + | CLI, TRM, TET, ERY, FUS, GEN, MUP, TEI, | lukD,lukE | *t*304 | CC5 | 6 | SCCmec_type_IVa(2B) | tet(38), blaI / blaR1 / blaZ, mecA, aph(3)’-III, | dfrB, fusA, gyrA, pbp4, | Inc18, rep3 |
| AA42 (Helsinki) | + | + | CLI, TRM, TET, DAP, ERY, FUS, MUP | lukD,lukE | *t*304 | CC5 | 6 | SCCmec_type_IVa(2B) | tet(38), blaI / blaR1 / blaZ, mecA | dfrB, fusA, grlA, gyrA, ileS, pbp2, pbp4, 23S | Inc18, rep4 |
| AA46 (Pietarsaari) | - | + | CLI, TRM, TET, DAP, ERY, FUS, GEN, MUP, RIF, TEI, VAN | lukD,lukE | *t*304 | CC5 | 6 | SCCmec_type_IVa(2B) | tet(38), blaI / blaR1 / blaZ, mecA | dfrB, fusA, grlA, gyrA, ileS, pbp2, pbp4, 23S | Inc18, rep5 |
| AA47 (Rovaniemi) | + | + | CLI, TRM, TET, ERY, FUS, MUP, RIF | lukD, lukE,lukF, lukS-PV | *t*008 | CC8 | 8 | SCCmec_type_IVa(2B) | tet(38), blaI / blaR1 / blaZ, mecA, mph(C) / msr(A), fosB, aph(3')-IIIa | gyrA, grlB, fusA, pbp4, | Inc18, repA_N, rep_trans(repUS7c) |
| AA49 (Tampere) | + | + | CLI, TRM, TET, DAP, ERY, FUS, GEN, MUP, RIF, TEI, | lukD, lukE,lukF, lukS-PV | *t*008 | CC8 | 8 | SCCmec_type_IVa(2B) | tet(38), blaI / blaR1 / blaZ, mecA, mph(C) / msr(A), fosB, aph(3')-IIIa | gyrA, grlB, fusA, pbp2, pbp4 | Inc18, repA_N, rep_trans(repUS7c) |
| AA57 (Rovaniemi) | + | + | CLI, TRM, TET, ERY, DAP, FUS, GEN, MUP, RIF | lukD, lukE,lukF, lukS-PV | *t*008 | CC8 | 8 | SCCmec_type_IVa(2B) | tet(38), blaI / blaR1 / blaZ, mecA, mph(C) / msr(A), fosB, aph(3')-IIIa | gyrA, grlB, fusA, pbp2, pbp4, 23S | Inc18, repA_N, rep_trans(repUS7c) |
| AA64 (Lappeenranta) | - | + | CLI, TRM, TET, DAP, ERY, FUS, GEN, MUP, RIF | lukD, lukE,lukF, lukS-PV | *t*008 | CC8 | 8 | SCCmec_type_IVa(2B) | tet(38), blaI / blaR1 / blaZ, mecA, mph(C) / msr(A), fosB, aph(3')-IIIa | gyrA, grlB, fusA, pbp2, pbp4, 23S | Inc18, repA_N, rep_trans(repUS7c) |
| AA66 (Pietarsaari) | + | + | CLI, TRM, TET, CPT, ERY, FUS, GEN, MUP, RIF, TEI, VAN | Nil | *t*172 | CC59 | 9068 | SCCmec_type_IVa(2B) | tet(38), blaI, blaI / blaZ, blaZ, mecA | gyrA, grlB, fusA, pbp2, pbp4, 23S | Inc18, repA_N, rep3(rep5a) |
| AA68 (Seinajoki) | - | + | CLI, TRM, TET, ERY, FUS, MUP, RIF | Nil | *t*034 | CC398 | 398 | SCCmec_type_IVa(2B) | tet(38) / tet(K) / tet(M), blaI / blaPC, blaR1, ant(9)-Ia, mecA, dfrG, lnu(B) / lsa(E) | gyrA, grlB, fusA, pbp2, pbp4, 23S | rep_trans (rep7a, repUS43) |
| AA70 (Turku) | - | + | CLI, TRM, TET, ERY, FUS, MUP | lukD,lukE | *t*304 | CC5 | 6 | SCCmec_type_IVa(2B) | tet(38), mecA | dfrB, gyrA, grlB, fusA, pbp2, pbp4, 23S | Nil |
| AA72 (Helsinki) | + | + | CLI, TRM, TET, DAP, ERY, FUS, GEN, MUP, RIF | lukD, lukE,lukF, lukS-PV | *t*008 | CC8 | 8 | SCCmec_type_IVa(2B) | tet(38), blaI/blaR1, blaZ, mecA, mph(C)/ msr(A), aph(3')-IIIa | dfrB, gyrA, grlB, fusA, pbp2, pbp4, 23S | Inc18, repA_N, rep_trans |
| AA75 (Oulu) | - | + | CLI, TRM, TET, DAP, ERY, FUS, GEN, MUP, RIF | lukD,lukE | *t*008 | CC8 | 8 | SCCmec_type_IVa(2B) | tet(38), blaI /blaR1/ blaZ, mecA, *sat*4, mph(C) / msr(A), fosB, aph(3')-IIIa | dfrB, gyrA, grlB, fusA, pbp2, pbp4, 23S | Inc18, repA_N |
